# Is the Mineral Content of Muscle Tissue (*Longissimus Lumborum*) in Cattle Finished During the Rainy Season in the Eastern Amazon Influenced by Different Farming Systems?

**DOI:** 10.3390/ani15152186

**Published:** 2025-07-25

**Authors:** Ana Paula Damasceno Ferreira, Jamile Andréa Rodrigues da Silva, Miguel Pedro Mourato, José António Mestre Prates, Thomaz Cyro Guimarães de Carvalho Rodrigues, André Guimarães Maciel e Silva, Andrea Viana da Cruz, Adriny dos Santos Miranda Lobato, Welligton Conceição da Silva, Elton Alex Corrêa da Silva, Antônio Marcos Quadros Cunha, Vanessa Vieira Lourenço-Costa, Éder Bruno Rebelo da Silva, Tatiane Silva Belo, José de Brito Lourenço-Júnior

**Affiliations:** 1 Postgraduate Program in Animal Science (PPGCAN), Institute of Veterinary Medicine, Federal University of Para (UFPA), Castanhal 68746-360, Brazil; anapaulaferreira0019@gmail.com (A.P.D.F.); andregms@gmail.com (A.G.M.e.S.); andrea.vianacruz@gmail.com (A.V.d.C.); adrinysantos2@gmail.com (A.d.S.M.L.); eltonpesc@gmail.com (E.A.C.d.S.); eder.b.rebelo@gmail.com (É.B.R.d.S.); tatianebelovet@gmail.com (T.S.B.); joselourencojr@yahoo.com.br (J.d.B.L.-J.); 2 Institute of Animal Health and Production, Federal Rural University of the Amazônia (UFRA), Belem 66077-830, Brazil; jamileandrea@yahoo.com.br (J.A.R.d.S.); thomazguimaraes@yahoo.com.br (T.C.G.d.C.R.); 3 Linking Landscape, Environment, Agriculture and Food (LEAF), Higher Institute of Agronomy, University of Lisbon, 1349-017 Lisboa, Portugal; mmourato@isa.ulisboa.pt; 4 Centre for Interdisciplinary Research in Animal Health (CIISA), Faculty of Veterinary Medicine, University of Lisbon, 1300-477 Lisboa, Portugal; japrates@fmv.ulisboa.pt; 5 Laboratório Associado para Ciência Animal e Veterinária (AL4AnimalS), Faculdade de Medicina Veterinária, Universidade de Lisboa, 1300-477 Lisboa, Portugal; 6 Nucleus of Agricultural Sciences and Rural Development, Federal University of Pará, Cametá 68400-000, Brazil; amqcunha@gmail.com; 7 Institute of Health Sciences, Federal University of Pará, Belem 66075-110, Brazil; vlourencocosta@hotmail.com

**Keywords:** Amazon, beef, minerals, extensive system, intensive system

## Abstract

The scientific literature currently lacks studies that evaluate the nutritional composition of the tissues of cattle raised in different systems, so that the nutritional effects can be known and used to enhance consumption and use in the diet. The aim was therefore to assess whether the mineral content of muscle tissue (*longissimus lumborum*) in cattle finished during the rainy season in the Eastern Amazon is influenced by different farming systems. The treatments consisted of four systems (three pasture production systems and one feedlot system). 1. native wetland pasture in Santa Cruz do Arari (Mesoregion of Marajó); 2. native wetland pasture in Monte Alegre (Mesoregion of Baixo Amazonas); 3. cultivated dryland pasture in São Miguel do Guamá (Mesoregion of Nordeste Paraense); and 4. Confinement in Santa Izabel do Pará (Metropolitan Region of Belém). The analyses were carried out on samples of the *longissimus lumborum* muscle tissue of 48 male, castrated, crossbred Nelore cattle, twelve per breeding system, from commercial farms, destined for meat production, finished during the rainiest period of the year (between January and June). In systems 1 and 2, the animals were slaughtered in licensed slaughterhouses; the animals in systems 3 and 4 were slaughtered in commercial slaughterhouses. Food sampling and chemical analysis, soil sample collection and analysis, *longissimus lumborum* muscle tissue collection, sample preparation and digestion, and inductively coupled plasma optical emission spectrometry were evaluated. The experimental design was completely randomized in a linear model with four rearing systems and one period (rainy). The data was compared using the Statistical Analysis Systems (SAS) program. All analyses were carried out considering a significance level of 0.05. Samples of the diets offered (pasture and concentrate) were also collected. The Amazon systems influenced the macro- and micromineral content in the muscles of cattle (*p* < 0.05). The interaction between pasture systems vs. confinement showed differences in the minerals calcium (Ca), magnesium (Mg), phosphorus (P), copper (Cu), zinc (Zn), iron (Fe), and manganese (Mn) (*p* < 0.05). However, there was no difference in the values of sodium (Na), potassium (K), and sulfur (S) between the rearing systems (*p* > 0.05). By contrast, the cultivated pasture system vs. extensive pasture showed differences in all the elements evaluated (*p* < 0.05). The rearing systems of the Eastern Amazon influenced the mineral content of beef, which continues to be an excellent source of macro- and microminerals and can compose the human diet.

## Simple Summary

The scientific literature currently lacks studies that evaluate the nutritional composition of the tissues of cattle raised in different systems, so that the nutritional effects can be known and used to maximize consumption and use in human diet. The aim was therefore to assess whether the mineral content of muscle tissue (*longissimus lumborum*) in cattle finished during the rainy season in the Eastern Amazon is influenced by different farming systems: 1. Native wetland pasture in Santa Cruz do Arari (Marajó Mesoregion); 2. Native wetland pasture in Monte Alegre (Lower Amazon Mesoregion); 3. Cultivated dryland pasture in São Miguel do Guamá (Northeast Pará Mesoregion); and 4. Confinement, in Santa Izabel do Pará (Mesoregion of Metropolitan Belém). The Amazonian systems influenced the macro and micromineral content in the muscle tissue of the cattle. The interaction between extensive pasture vs. extensive feedlot systems showed differences in the minerals calcium (Ca), magnesium (Mg), phosphorus (P), copper (Cu), zinc (Zn), iron (Fe) and manganese (Mn). In this way, the farming systems in the Eastern Amazon influence the mineral content of beef, which remains an excellent source of macro- and microminerals and can be part of the human diet.

## 1. Introduction

Some foods, such as beef, have variable compositions because they are obtained from genetically superior animals, with different ages, nutritional management, and rearing systems [1,2]. In Brazil, due to its large territorial extension and wide edaphoclimatic variety, we have numerous breeds, chosen for their adaptability, and rearing systems that are adapted to the conditions of each location. These characteristics favour the variation in the composition of the final product of national beef cattle farming, especially minerals.

In the state of Pará, located in the Amazon biome, beef cattle farming is developed mainly in pasture systems, such as the native pastures of Marajó, Baixo, and Médio Amazonas and cultivated pastures using forages with better nutritional value [3]. These play a relevant role in the development of livestock and social and economic development in the region, as they are ecologically stable ecosystems [4,5]. In addition to these, confinement has been standing out, with high growth in recent years, and is a strategy for better use of land and greater efficiency of animals [6].

Regardless of the breeding system, adequate nutrition is a determining factor for the health and performance of animals and should provide, among other things, adequate levels of macro- and microminerals [7]. Therefore, the assessment of mineral concentration becomes important since, in addition to providing information about the composition of soil and forage, it optimizes the formulation of diets for humans [8]. Techniques, such as inductively coupled plasma optical emission spectrometry (ICP-OES), have been used to investigate the absolute concentrations of elements in animal tissues, as performed by Rodrigues et al. [3], who evaluated the mineral profile in the muscle tissue of buffaloes raised in the Eastern Amazon. Considerable differences in the mineral composition in all bovine tissue, depending on the geographic location. De Freitas et al. [9] found variations in the mineral composition of the *longissimus dorsi* muscle tissue in steers from different genetic groups finished in feedlots or on pasture.

The scientific literature currently lacks studies that evaluate the nutritional composition of tissues from cattle raised in systems in the Eastern Amazon, which makes this research unprecedented for the beef production chain in the state of Pará. In this context, we hypothesize that the main rearing systems in the Eastern Amazon influence the mineral content of beef. The aim was therefore to assess whether the mineral content of muscle tissue (*longissimus lumborum*) in cattle finished during the rainy season in the Eastern Amazon is influenced by different farming systems.

## 2. Materials and Methods

The work was exempted from formal analysis by the Animal Use Ethics Committee (CEUA), of the Federal Rural University of the Amazon (UFRA), Belém, Pará, Brazil, through protocol no. 1928240123, dated 1 August 2023, considering the use of slaughtered animals. The experimental design was completely randomized.

### 2.1. Production Systems and Diets

**Figure 1:**
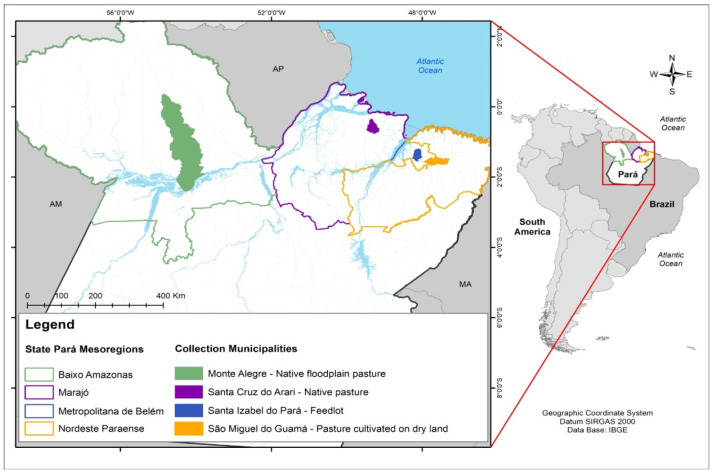
Geographical distribution of the main cattle breeding systems for meat production in the state of Pará (Eastern Amazon).

The treatments consisted of four systems (three pasture production systems and one feedlot system) (Figure 1):

1. Native pasture of floodable lands in Santa Cruz do Arari, (mesoregion of Marajó SCA), the cattle in this system were raised extensively with feed based on native forages from floodable areas in continuous grazing, and water intake “ad libitum”. The average weight at slaughter was 410 kg at an age of 36 months.
2. Lowland native pasture in Monte Alegre (Lower Amazon mesoregion MA), the type of breeding in this system was exclusively in lowland and upland native pasture called “covered fields”. At slaughter, the cattle weighed an average of 450 kg and were 36 months old.
3. Pasture cultivated on terra firma in São Miguel do Guamá (Northeast Pará Mesoregion—SMG), this breeding system worked with the rearing and finishing of beef cattle in the rainy season in a total area of 160 hectares in paddocks formed by *Panicum maximum cv.* In rotational grazing, the entry and exit of the animals were determined by the height of the forage, 20 kg of palm oil concentrate (*Elaeis guineensis*) and protein nucleus were also supplied per animal/day. The intake of clean and fresh water was ad libitum. The lot of crossbred Nelore cattle slaughtered from this system was homogeneous with an average weight of 550 kg at 24 months.
4. Intensive confinement system in Santa Izabel do Pará (Metropolitan Mesoregion of Belém—SI). In the feedlot, only the finishing phase of the cattle was worked on in a total area of 943 hectares, the finishing period of these animals was 96 days, and the average daily weight gain was 1.635 kg. The entry weight of the animals in the feedlot was 464.6 kg, and the exit weight was 628.7 kg. The average carcass weight of the slaughtered animals was 341 kg with 55.81% yield. The diet was feed for 135 days, consisting of soybean meal, barley, *Mombaça grass* silage, corn meal, cassava husks, urea, and high-performance core (Table 1 and Table 2).

### 2.2. Animals and Samples

The analyses were carried out on *longissimus lumborum muscle tissue samples* from 48 male cattle, castrated, crossbred of the Nellore breed, twelve by the breeding system, originating from commercial farms, intended for meat production, finished during the rainiest period of the year (between January and June). In systems 1 and 2, the animals were slaughtered in licensed slaughterhouses, and the animals in systems 3 and 4 were slaughtered in commercial slaughterhouses.

### 2.3. Food Sampling and Chemical Analysis

The collection of ingredients (concentrate) and pasture followed the methodology used by Silva et al. [10]. The chemical analyses all followed the INCT-CA methodology: The total nitrogen—NT contents were determined by the N001/1 methodology through micro Kjeldhal to obtain crude protein (CP). The ashes, method M-001/1, in a muffle furnace at 600 °C, for four hours. Ether Extract (EE) method G-004/1. Neutral detergent fiber (NDF), according to methodologies F-001/1 and F-002/1. Acid detergent fiber (ADF), following INCT-AC F-003/1 and INCT-AC F-004/1. In the last two, there was a correction for proteins and ashes (recommended by the National Institute of Science and Technology in Animal Science). Non-fiber carbohydrates (NFC), according to the methodology described by Sniffen. Total digestible nutrients (TDN), according to the Clemson University equation: TDN = 93.59 − (FDA × 0.936).

### 2.4. Collection and Analysis of Soil Samples

Soil samples were collected according to Embrapa [11] and analyzed at the Brazilian Institute of Analysis—IBRA. The following were evaluated: physical and chemical profile of the soil; determination of macro- (Ca, Mg, K, P, S, Na) and trace minerals (Co, Zn, F, Mn, Br); soil organic matter and acidity based on the methods IAC—Chemical Analysis for the Evaluation of Tropical Soil Fertility, Agronomic Institute of Campinas, (2001) and Embrapa—Chemical Analysis for the Evaluation of Soil Fertility—EMBRAPA, prepared by Silva et al. [12].

### 2.5. Longissimus Lumborum Muscle Tissue Collection

Tissue collections took place in commercial slaughterhouses for animals from herds of meat production and commercialization. From each animal, 100 g (wet weight) were collected, which were ground in an electric meat grinder and freeze-dried for 48 h at a temperature of −42 °C and −45 °C and pressure of 158 μm Hg up to 256 μm Hg, uninterrupted, in a freeze dryer (LIOTOP, L101, Liobraz^®^, São Paulo, Brazil) at the Animal Nutrition Laboratory of the Federal Rural University of Amazon, Belém, Para. The lyophilized samples were processed in an automatic food grinder, vacuum-packed, and sent to the Instituto Superior de Agronomia (ISA, School of Agronomy, UL) of the University of Lisbon, Portugal, for analysis.

### 2.6. Mineral Analysis

#### 2.6.1. Sample Preparation and Digestion

Muscle tissue samples (0.3 g) were weighed in digestion tubes (50 mL) for analysis in a simple sampling method. The dissolution of the samples was carried out according to the methods of Roselli et al. [13] and Ribeiro et al. [14]. An amount of 3 mL of concentrated nitric acid and 10 mL of hydrochloric acid were added to each digestion tube, remaining submerged for 16 h. After this period, 1 mL of hydrogen peroxide was added before digestion to avoid sample loss due to the reaction between them. After the addition of the acidic mixture, the tubes were randomly distributed on a digestion plate (DigiPREP MS, SCP Science, Analytichem^®^, Montreal, QC, Canada), and heated for 1 h to reach 95 °C and then additionally for 1 h at 95 °C, following the 137 standards. After a total digestion time of two hours, the samples were cooled in a ventilated chamber. Upon reaching room temperature, the samples were diluted with distilled water in a volumetric flask (25 mL). The diluted samples were filtered in sealed flasks with 90 mm diameter filter paper (Filter-Lab^®^ ref. 1242, FILTROS ANOIA S.A., Barcelona, Spain). Part of the filtered solution was transferred to ICP tubes, and then they were organized on a conveyor. Each conveyor corresponds to a digestion set.

#### 2.6.2. Inductively Coupled Plasma Optical Emission Spectrometry

The ICP-OES readings were performed in a Thermo Scientific iCAP 7000 series, Ontario, CA, Canada, ICP-OES spectrometer equipped with an autosampler. Multi-element patterns were used to create the calibration curves needed to quantify the different elements. The following were detected and quantified: Zn (zinc), Fe (iron), Mn (manganese), Cu (copper), S (sulfur), P (phosphorus), Mg (magnesium), Ca (calcium), K (potassium), and Na (sodium) [14].

### 2.7. Statistical Analysis

In this study, the sample size was 40 animals, 10 animals from each breeding system. We consider the treatment to be the rearing system and the animals to be the repetitions. The experimental design was completely randomized in a linear model with four rearing systems and one (rainy) period. The data were compared using the Statistical Analyses Systems program (SAS version 9.1.3; SAS Institute, Inc., Cary, NC, USA). Both use the general linear model (GLM PROC). Contrast analysis was used to test differences between the means of groups in a statistical model. The analysis tested systems 1, 2, and 3 to system 4, and systems 1 and 2 to system 3. To test the differences between the four systems, an analysis of variance (ANOVA) was performed [15], followed by Tukey’s test of comparison of means. A principal component analysis (PCA) was performed and the standardization was performed using the *scale* function, using the factoMineR package, and the visualization of the biplot using the factoextra package, in the R studio version 4.3.2 program [16]. All analyses were performed considering a significance level of 0.05 [17].

## 3. Results

The chemical and mineral composition of the diets fed to crossbred Nelore cattle raised in systems in the Eastern Amazon are elucidated in Table 3. Table 4 presents the data from the soil analyses of the different pasture systems in the Eastern Amazon.

### 3.1. Statistical Values of Mineral Concentration in the Bovine Muscle Tissue of Four Rearing Systems

Table 5 illustrates the differences in the mineral composition of the tissues. The rearing systems of the Eastern Amazon statistically influenced the mineral content in the muscle tissue of crossbred Nelore cattle (mg/kg). Sodium (5216 mg/kg) and calcium (1020.2 mg/kg) values were higher in the extensive system (*p* < 0.01). Similarly, the cultivated pasture system was superior in São Miguel do Guamá, SMG, for the mineral elements potassium (15,232.34 mg/kg), magnesium (712.74 mg/kg), and phosphorus (8935.31 mg/kg) (*p* < 0.01). The sulfur content was high in the pasture systems with a small variation of (8520.47–8281.50 mg/kg), as shown in Table 5.

The extensive systems exhibited higher levels of trace minerals. For example, copper (7.25 mg/kg) and manganese (3.6 mg/kg) were superior in the native pasture system, SCA (*p* < 0.01). The extensive system revealed higher iron concentrations ranging from (149.19–138.77 mg/kg), as well as zinc content (278.82 mg/kg), which was higher in native pasture, MA.

### 3.2. Statistical Evaluation Among Cattle Rearing Systems in Eastern Amazonia

In the direct evaluation between the pasture systems and the intensive system, there was a difference in the macrominerals Ca, Mg, and P and trace minerals as shown in Table 5. The elements Na (*p* = 0.15), K (*p* = 0.58) and S (*p* = 0.33) were statistically insignificant. The microminerals showed a difference in the contrast between the pasture and feedlot systems (*p* < 0.01). The animal tissue samples from the extensive systems were highlighted by elements such as zinc and iron, as well as the copper and manganese content.

The correlation between the extensive systems (ACS and MA) and the cultivated pasture system (SMG) showed a difference for all mineral elements evaluated in the muscle tissue of cattle (*p* < 0.01). The concentrations of minerals (K, P, Mg, and S) were strongly influenced in a cultivated pasture system (SMG). Other elements (Na, Ca, Cu, Mn, and Fe) showed high values in an extensive system, SCA, leaving only Zn and Fe that stood out in MA cattle.

### 3.3. Principal Component Analysis

**Figure 2:**
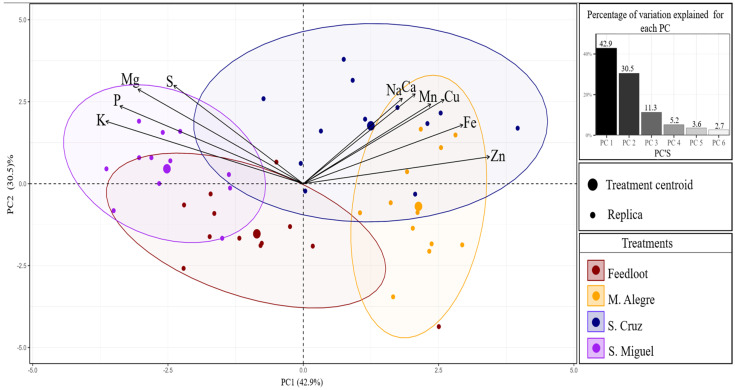
PCA chart for macro and trace minerals in beef.

The first two principal components of principal component analysis (Figure 2) retained 73.4% of the variability in the macromineral and micromineral data. PC1 explained 42.9% of the variation, presenting in its positive portion the trace minerals Zn, Fe, Cu, and Mn. In the negative portion of PC1, the macrominerals K, P, Mg, and S were projected. PC2 explained 30.5% of the variation in the data, wherein the positive portion of the macrominerals S, Mg, Ca, Na, and P, and the microminerals Cu and Mn were projected.

The area of influence of the ellipses indicated differences in the composition of the minerals between the treatments. The main variation occurred between the extensive system of Monte Alegre, which is in the positive portion of PC1, and the extensive system of São Miguel do Guamá, which is isolated in the negative portion of PC1.

## 4. Discussion

### 4.1. Characterization of Mineral Content in Four Rearing Systems

In the characterization of the four breeding systems, the composition of macro- and microminerals showed significant differences. This result can be attributed to the differences between the systems, since mineral status can be influenced by edaphoclimatic characteristics, geographic location, nutrition, management practice, and genetics [14].

The evaluations showed that the mineral content in muscle tissue (*longissimus lumborum*) of cattle raised in the pasture system was strongly influenced, showing higher averages of mineral content. Reports infer that the fat–mineral ratio in the tissues of young cattle finished with forage and grains results in greater fat deposition that dilutes mineral concentrations, because there is a lower concentration of minerals in fat than in muscle [18]. Such suggestion in the scientific literature is in line with weight and age, since the animals that received a concentrate-based diet were slaughtered with a higher weight in a shorter period of time, with higher carcass yields that also presented greater fat conformation, reaffirming the assumption in the evaluation of the results of mineral content of animals raised in an intensive system.

Another conjecture suggests that grazing animals during the rainy season have access to forage in abundance, while in the dry season there is a lower supply of forage. Fluctuations in food quality can result in mineral imbalances in tissues over the years, being even more critical in the dry season, due to the lignification of natural pastures and water scarcity [14]. As the plant matures, its mineral content declines due to a natural process of dilution and translocation of nutrients to the root system [19], the diversity of soils in the Eastern Amazon also contributes to different fertility of minerals that will be absorbed by the plants.

The concentrations of macrominerals Na and Ca were more evident in muscle tissue samples of the extensive system, SCA. The high sodium content can potentially be related to the feeding of native pastures in poorly drained savannas, a common condition in Marajó, where the waters exhibit considerable levels of sodium, being classified as brackish water [20,21]; such investigations are structured with the characteristics of the system, mainly with the result of 1.5% of this mineral in the soil CEC that is considered in the literature to be above 1% high [22].

Sodium levels in edible tissues have been previously evaluated in different research. Araújo et al. [23], evaluated the mineral profile of sheep meat, with different water supplies, in Pernambuco, Brazil, and observed that the supply of 40% of water had a positive association between all minerals evaluated, highlighting a strong and positive correlation for Na contents. Rodrigues et al. [3], who evaluated the mineral profile of buffaloes raised in an extensive ecosystem of Marajó, found high sodium content in the muscle tissue (*longissimus lumborum*) of buffaloes (*Bubalus bubalis*).

Despite the high levels of sodium in the muscle tissue of cattle from extensive systems, it is important to emphasize the need for adjustments to the daily intake of this food as recommended by a human nutrition professional.

In a study evaluating the Ca levels of poorly drained savanna pastures on the island of Marajó, Sá et al. [20] concluded that the minimum requirements of beef animals were met, which presented an average of 2500 mg/kg (DM). Ca levels showed the highest average (1020.24 mg/kg) in cattle raised extensively in Santa Cruz do Arari. Lower concentrations in this study were (SD = 619.21 mg/kg; RS = 520.29 mg/kg) in samples of *longissimus* muscle tissue from buffaloes in a native pasture system in Marajó found by Rodrigues et al. [3]. Forages are considered good sources of calcium, and their concentration can vary with the species, portion, maturity of the plant, and the amount of exchangeable calcium in the soil and climate [24]. A fact that should be observed is that, while the concentrations of certain minerals such as P, K, Mg, Na, Cu, Fe, and Zn decrease with the advance of the age of the plant, the concentration of Ca remains almost unchanged, which can favour antagonistic processes and the use of other minerals in the plants.

Since the mineral profile of meat is a complex characteristic and is influenced by environmental factors [25], it is admitted that the extensive system, ACS, influenced the result in question. Animals fed concentrate-based diets, rich in phosphorus, tend to have lower concentrations of calcium in their tissues, as observed in cattle fed in an intensive system. This was also observed by Pilarczyk [26], who analyzed the calcium content and noticed a variation of 157.6 and 163.1 mg/kg in samples of cattle of different breeds raised in a feedlot. This result could be attributed to the imbalance of nutrients in the diet that inhibited calcium absorption due to high levels of P.

The mineral element sulfur was the only one among the macrominerals with a subtle variation of (8520.47–8281.50 mg/kg) between the pasture systems of Santa Cruz do Arari and São Miguel do Guamá. Concentrations of this element in the food of both systems also exhibit approximate values, which may have elucidated the data. The concentration of other macrominerals, such as Mg, K, and P, were higher in cultivated pasture systems. The magnesium result of this study is close to the levels found by Santana et al. [27] in different muscles of cattle fed on pastures in different regions in the state of Bahia, Brazil, which exhibited variation (362.3–551.3 mg/kg) of magnesium. Like Freitas et al. (2014), they also observed higher values in *longissimus dorsi* muscle tissue samples from animals in pasture systems when compared to those in feedlots. These concentrations confirm its potential since the average values indicated for daily consumption range from 310 to 320 mg for women and from 400 to 420 mg for adult men [28].

Frequently, young forages have high concentrations of potassium, and the level of K is an important risk factor in the development of tetany, mainly because it decreases magnesium absorption, which can trigger hypomagnesemia [29]. This important verification possibly affected the potassium concentration, which remained high in tissue samples and in the feed consumed at pasture (16,267.8 mg/kg) and palm oil sludge by-product (8164 mg/kg). Possibly another factor that may have influenced the K content was the area formed of cultivated pasture of terra firma in São Miguel do Guamá that received potassium and phosphate fertilization, in addition to the favourable conditions during the rainy season, which provided leaf growth of the plant.

The phosphorus content, which was also higher in animals from this system, may have been influenced by the P concentrations in the pasture of Panicum maximum cv Mombaça (2108.4 mg/kg). Rodrigues et al. [3] analyzed the P content in the pasture of the same cultivar of this system, implemented on a property in Nova Timboteua in the Eastern Amazon that served as food for buffaloes and obtained an approximate result (2426.34 mg/kg) to that of this study. Santana et al. [27] found a variation of phosphorus (3270–10,830 mg/kg) in different tissues of cattle raised in Bahia, Brazil.

The trace minerals Cu, Zn, Fe, and Mn statistically exhibited higher concentrations in crossbred Nelore muscle tissue reared in extensive systems of Monte Alegre and Santa Cruz do Arari. The soil–plant–animal interface can exemplify what happens in unique ecosystems of the Eastern Amazon where activities such as beef cattle ranching are developed, which takes advantage of the use of natural resources such as cattle raising in native pastures of natural fields. The so-called “Amazon Dark Earth” are floodplain areas or areas that suffer constant flooding throughout the year, characterized by high concentrations of calcium, magnesium, zinc, and manganese cations [30,31]. These allow the occurrence of hydromorphic soils of the gley type (humic and low humic); in general, they are of high acidity and clayey texture, with a high percentage of organic matter, as shown in Table 4. The fertility of floodplain soils is concentrated in its superficial layer, due to the incorporation of nutrients released by organic matter from the decomposition of forest plant material, as well as the deposition on the soil of mineral and organic substances suspended in the muddy waters of the Amazon River, caused by tidal movement, generating the high fertility of floodplain soils [32].

In addition, studies suggest that the muscles involved in movements have high oxidative metabolism and are highly concentrated in Zn and Fe. Cattle raised on pasture tend to perform more movements because they are more selective in the search for aerial parts of the leaves, less lignified portions and with better quality. Such evidence may explain the high concentrations of these minerals in the muscle tissue of cattle in the extensive systems of this study [32,33,34]

The concentration of Cu found in the meat of animals from the Eastern Amazon, Pará systems was high in animals fed on pastures, especially Santa Cruz do Arari (7.25 mg/kg). It is possible this result reflects the level of Cu present in the pasture, which presented a value of (6.19 mg/kg). The minimum requirement for beef cattle nutrition is 4 mg/kg of forage dry mass copper [35].

The scientific literature lacks studies that investigate the composition of minerals in different animal husbandry systems in the Amazonian context during the rainy season, emphasizing the importance of this study that fosters future research of this nature. These investigations contribute to explanations about the differences and variations that can affect the mineral content in the muscle tissue, being an auxiliary tool for the formulation of human diets with greater accuracy, since fresh foods, such as beef, are recommended in the Food Guide of the Brazilian Population [36].

Among the few resources found in the bibliography, some studies stand out, such as those by Rodrigues et al. [3] who found low Fe concentration in samples of buffaloes raised in extensive and intensive systems in the Eastern Amazon, and that of Freitas et al. [9] who observed high levels of Zn in the muscle tissue of Hereford and Braford cattle fed on pasture in southern Brazil. The absorption of mineral elements, along with transport, storage and excretion are steps in a dynamic system that maintains normal food intake, resulting in homeostasis. The absorption of copper and iron may be low, due to the low solubility in the anaerobic environment of the rumen [37].

Minerals in the muscles of cattle play a crucial role in the nutritional quality of the meat, as they affect several biological processes in the animals and some characteristics, such as colour and texture [38].

### 4.2. Comparison Between Cattle Breeding Systems in Eastern Amazon

The comparison between pasture vs. feedlot systems revealed significant differences for almost all minerals except for sodium, potassium, and sulfur. Possibly these minerals were in proportional concentrations in the animals’ diet since the amount of sodium incorporated in the total diet can be in a proportion of 30% to 50% because it is palatable and well accepted; this makes it an important vehicle for the intake of other minerals [39].

However, when the pasture-rearing systems were directly compared, all the elements were statistically different. Based on statistical data, it is assumed that the ecosystems of the Eastern Amazon are distinct and that they have peculiarities that can impact the composition of minerals in edible tissues intended for meat production, especially in cattle-raising systems on pastures.

Minerals have a great influence on animal metabolism and human health, with a wide variety of metabolic functions, such as enzymatic function, osmotic pressure control, and muscle contraction [40,41]. Macrominerals are required in amounts of at least 100 mg/day and are found mainly in an ionic state, both in food and in the body, constituting around 0.01% of total body weight [42,43]. Given this, it can be stated that the concentrations of minerals observed in the *longissimus lumborum* muscle tissue, regardless of the rearing system, are by the standards recommended for human consumption. It is an important food in the prevention of diseases caused by the deficiency of certain minerals, such as anaemia, a condition accompanied by fatigue, pallor, lack of appetite, thermoregulation problems, and weakening of the immune system [44].

It was clear that the native pastures of SCA and MA were more efficient with higher levels of minerals, especially iron, zinc, copper, and manganese, than the other systems. In the same vein, but with other minerals standing out, SMG had higher levels of potassium, magnesium, and phosphorus.

## 5. Conclusions

The edible tissue originating from cattle production in the Eastern Amazon has good amounts of mineral nutrients regardless of the rearing system, especially K, P, S, Zn, and Fe. The results underscore the importance of understanding the complex interplay between the ecosystem characteristics that influence the composition and mineral absorption of animals raised in the Amazon biome. However, more research must be carried out in this domain to accurately assist field professionals who work in the beef chain in the Amazon context, in addition to the importance of minerals in the formation of tissues. The uniqueness of breeding environments must be respected so that a conscious and sustainable livestock can be developed, in addition to being an important tool for developing a healthy and balanced diet for humans.

## Figures and Tables

**Table 1:** Characterization of breeding systems.

Item	System, Pará, Eastern Amazon			
	Native Pasture	Native Pasture	Cultivated Pasture	Confinement
Location	Monte Alegre	Santa Cruz do Arari	São Miguel do Guamá	Santa Izabel
Climate (Kõppen–Geiger)	* Am	* Am	* Am	** Af
Precipitation (mm)	1.780	2.500	2.250	2.599
Temperature (media/annual)	27	26	26	26
RH% (media/annual)	72	86	85	85
Rainy season	December/May January/June	December/May January/June	January/June	January/June
Dry season	August/October	September/November	September/November	July/December

Note: RH% = Relative humidity. * Am is the code for monsoon climate. ** Af is the code for the equatorial climate.

**Table 2:** Ingredients and proportion of the diet of cattle raised in intensive systems.

Ingredients	Proportion (%)	% DM	Quantity (kg)
Cassava husk	15	38.33	39.13
Barley	8	27.67	28.91
Corn	60	88.00	68.18
Premix	8.41	99.00	8.49
Grass silage	8.59	31.66	27.13
Total	100	-	171.85

Note: % DM—dry matter.

**Table 3:** Chemical and mineral composition of food.

	Pasture				Confinement
	SCA	MA	SMG		SI
Item (%)	Native Pasture	Native Pasture	Cultivated Pasture	Palm Oil Linters	Total Diet
DM	39.14	21.33	29.75	43.45	54.57
OM	91.92	90.45	94.53	74.99	89.92
CP	11.75	7.69	8.17	29.67	12.72
EE	2.23	1.59	1.50	6.32	3.37
NDF	73.64	73.40	74.27	35.15	30.72
ADF	25.23	27.53	26.83	7.20	11.26
TDN	69.98	67.82	46.90	86.85	79.55
NFC	4.30	7.77	10.59	3.85	43.11
Ash	8.08	9.55	5.47	25.01	10.08
Minerals (mg/kg/ms)					
Na	2460.2	2252.9	2240.2	17,740.5	3296.5
K	21,943.9	14,528.9	16,267.8	8164.0	4164.2
Ca	3722.4	2598.5	5217.1	39,970.0	7011.8
Mg	1337.8	1889.3	2675.6	4116.0	2033.9
P	1286.3	524.0	2108.4	2670.7	2027.6
S	1866.3	1076.2	1211.9	4514.8	1503.1
Cu	6.19	9.05	7.52	59.11	14.50
Zn	45.84	37.17	23.82	170.9	74.44
Fe	659.1	830.79	95.13	3787.5	545.7
Mn	295.8	174.91	51.89	410.0	86.95

Note: SCA = Santa Cruz do Arari; MA = Monte Alegre; SMG = São Miguel do Guamá; SI = Santa Izabel do Pará; DM—dry matter; OM—organic matter; CP—crude protein; EE—ethereal extract; NDF—neutral detergent fiber; ADF—acid detergent fiber; TDN—total digestible nutrients; NFC—non-fiber carbohydrates; ashes.

**Table 4:** Soil composition in the breeding systems of the Amazon (Brazil) during the rainy season of the year.

Item (%)	Breeding Systems		
	Santa Cruz do Arari	Monte Alegre	São Miguel do Guamá
pH	4.1	4.1	4.6
M.O g/dm^3^	24	29	20
P ^1^	3.5	6	9
S ^1^	24	6	9
Cu ^1^	0.3	2.6	0.6
Fe ^1^	54	1600	165
Zn ^1^	5.3	2	1.4
Mn ^1^	28	4.4	8.6
K ^2^	0.3	1.7	1.4
Ca ^2^	6.6	3.7	37.3
Mg ^2^	19.8	1.9	7.8
Na ^2^	1.5	0.6	0.6
Al ^2^	33	16.8	0
H + Al ^3^	207	99	27
C.T.C ^3^	288.1	107.4	51
Argila g/kg	569	328	127

Note: ^1^ mg/dm^3^, ^2^ in CEC (%), ^3^ mmol/dm^3^; MO—organic matter; Al—exchangeable aluminum; H + Al3—total acidity; C.T.C—Cation exchange capacity; Na, sodium; K, potassium; Ca, calcium; Mg, magnesium; P, phosphorus; S, sulfur; Cu, copper; Zn, zinc; Fe, iron; Mn, manganese.

**Table 5:** Mineral composition of muscle tissue from Nelore cattle intensively and extensively finished in pasture systems and a feedlot in the Amazon (Brazil) in the rainy season.

		Extensive System (SE)		Intensive System (SI)	*p*-Values			
	SCA ^1^	MA ^2^	SMG ^3^	Confinement ^4^	EP	Systems	Pasture vs. Pasture Confinement	Extensive vs. Extensive Cultivated Pasture
Macromineral (mg/kg/ms)								
Na	5216.00 ± 786.3 ^a^	4183.39 ± 318.2 ^b^	3900.77 ± 342.2 ^b^	4201.31 ± 278.7 ^b^	124.5	<0.01	0.15	<0.01
K	13,679.56 ± 1147 ^b^	12,430.74 ± 651.3 ^c^	15,232.34 ± 601.7 ^a^	13,947.21 ± 1072 ^b^	250.5	<0.01	0.58	<0.01
Ca	1020.24 ± 216.9 ^a^	824.73 ± 117.5 ^b^	733.33 ± 101.3 ^b^	711.78 ± 82.4 ^b^	37.4	<0.01	<0.01	<0.01
Mg	629.04 ± 77.4 ^b^	498.06 ± 61.7 ^c^	712.74 ± 58.2 ^a^	557.90 ± 92.6 ^bc^	20.9	<0.01	0.03	<0.01
P	7848.61 ± 645.2 ^b^	7272.14 ± 392.2 ^c^	8935.31 ± 422.1 ^a^	7510.37 ± 607.8 ^bc^	149.8	<0.01	0.01	<0.01
S	8281.50 ± 320.4 ^ab^	7796.61 ± 474.0 ^c^	8520.47 ± 363.7 ^a^	8060.00 ± 501.3 ^bc^	119.8	<0.01	0.33	<0.01
Micromineral								
Cu	7.25 ± 0.8 ^a^	6.84 ± 0.4 ^a^	6.08 ± 0.3 ^b^	4.86 ± 0.2 ^c^	0.13	<0.01	<0.01	<0.01
Zn	241.45 ± 21.8 ^b^	278.82 ± 36.4 ^a^	170.73 ± 19.0 ^c^	197.93 ± 24.1 ^c^	8.93	<0.01	<0.01	<0.01
Fe	138.77 ± 19.1 ^a^	149.19 ± 24.4 ^a^	115.05 ± 9.1 ^b^	104.74 ± 14.7 ^b^	7.23	<0.01	<0.01	<0.01
Mn	3.60 ± 0.5 ^a^	3.17 ± 0.3 ^ab^	2.79 ± 0.2 ^b^	2.89 ± 0.5 ^b^	0.11	<0.01	0.04	<0.01

Note: Values with different superscripts indicate significant differences when the interaction is significant (*p* < 0.05). Abbreviations: ^1^ SCA = Santa Cruz do Arari; ^2^ MA = Monte Alegre; ^3^ SMG = São Miguel do Guamá; ^4^ Na, sodium; K, potassium; Ca, calcium; Mg, magnesium; P, phosphorus; S, sulfur; Cu, copper; Zn, zinc; Fe, iron; Mn, manganese. EP: Standard error.

## Data Availability

The data presented in this study are available upon reasonable request from the corresponding author.

## References

[1] Lin K.C., Cross H.R., Johnson H.K., Breidenstein B.C., Randecker V., Field R.A. (1988). Mineral Composition of Lamb Carcasses from the United States and New Zealand. Meat Sci..

[2] Taniguchi C.N., Dobbs J., Dunn M.A. (2017). Heme iron. non-heme iron, and mineral content of blood clams (*Anadara* spp.) compared to *Manila clams* (*V. philippinarum*), *Pacific oysters* (*C. gigas*), and beef liver (*B. taurus*). J. Food Compos. Anal..

[3] Rodrigues L.S., Silva J.A.R., Lourenço-Júnior J.B., Silva A.G.M., Almeida A.M., Mourato M.P., de Castro V.C.G., Bezerra A.S., da Silva W.C., Prates J.A.M. (2023). Muscle mineral profile of water buffaloes (*Bubalus bubalis*) reared in different production systems of the Brazilian Eastern Amazon. Front. Vet. Sci..

[4] Townsend C.R., Costa N.D.L., Pereira R.D.A. (2012). Pastagens Nativas da Amazônia Brasileira.

[5] Rodrigues L.S., Silva J.A.R., Lourenço-Júnior J.B., Silva A.G.M., Almeida A.M., Mourato M.P., de Castro V.C.G., da Silva W.C., Prates J.A.M. (2023). Mineral content of liver of buffaloes (*Bubalus bubalis*) reared in different ecosystems in the eastern Amazon. Animals.

[6] Lanna D.P.D., de Almeida R. (2005). A terminação de bovinos em confinamento. Visão Agrícola..

[7] Organização Pan-Americana da Saúde (OPAS) (2019). Folha Informativa Alimentação Saudável. https://www.paho.org/bra/index.php?option=com_content&view=article&id=5964:folha-informativa-alimentacao-saudavel&Itemid=839.

[8] Ribeiro D.M., Scanlon T., Kilminster T., Martins C.F., Greeff J., Milton J. (2020). Mineral profiling of muscle and hepatic tissues of Australian Merino, Damara and Dorper lambs: Effect of weight loss. J. Anim. Physiol. Anim. Nutr..

[9] De Freitas A.K., Lobato J.F.P., Cardoso L.L., Tarouco J.U., Vieira R.M., Dillenburg D.R., Castro I. (2014). Nutritional composition of the meat of Hereford and Braford steers finished on pastures or in a feedlot in southern Brazil. Meat Sci..

[10] Silva J.A.R., Rodrigues L.S., Lourenço-Júnior J.B., Alfaia C.M., Costa M.M., Castro V.C.G. (2022). Total lipids, fatty acid composition, total cholesterol and lipid-soluble antioxidant vitamins in the longissimus lumborum muscle of water buffalo (*Bubalus bubalis*) from different production systems of the Brazilian Eastern Amazon. Animals.

[11] Embrapa Amapá (2012). Coleta de Solo Para Análise: Orientações.

[12] Silva F.D., Eira P.D., Barreto W.D.O., Perez D.V., Silva C.A. (1998). Análises Químicas para Avaliação da Fertilidade do solo: Métodos Usados na Embrapa Solos.

[13] Roselli C., Desideri D., Ma M., Fagiolino I., Feduzi L. (2016). Essential and toxic elements in meat of wild birds. J. Toxicol. Environ. Health..

[14] Ribeiro D.M., Mourato M.P., Almeida A.M. (2019). Assessing mineral status in edible tissues of domestic and game animals: A review with a special emphasis in tropical regions. Trop. Anim. Health Prod..

[15] Kutner M.H., Nachtsheim C.J., Neter J., Li W. (2005). Applied Linear Statistical Models.

[16] Kassambara A. (2017). Practical Guide to Cluster Analysis in R: Unsupervised Machine Learning.

[17] Tukey J.W. (1991). The philosophy of multiple comparisons. Stat. Sci..

[18] Williams J.E., Wagner D.G., Walters L.E., Horn G.W., Waller G.R., Sims P.L., Guenther J.J. (1983). Effect of Production Systems on Performance, Body Compostion and Lipid and Mineral Profiles of Soft Tissue in Cattle. J. Anim. Sci..

[19] McDowell L.R. (1999). Minerais para Ruminantes sob Pastejo em Regiões Tropicais Enfatizando o Brasil.

[20] Sá T.D.D.A., Móller M.R., Camarão A.P. (1998). Teores de Minerais em Pastagens Nativas de Savanas mal Drenadas da Ilha de Marajó, Pará.

[21] Müller J. (2020). Mosaico Temático Volume 3.

[22] Sobral L.F., Barretto M.C.D.V., Silva A.J.D., Anjos J.L.D. (2015). Guia Prático para Interpretação de Resultados de Análises de Solos.

[23] Araújo A.C., Magalhães A.L.R., Araújo G.G.L., Campos F.S., Gois G.C., Santos K.C. (2023). Correlation between mineral profile, physical-chemical characteristics, and proximate composition of meat from Santa Ines ewes under water restriction. Semin. Ciênc. Agrar.

[24] Minson D.J. (1990). Forages in Ruminant Nutrition.

[25] Diniz W.J., Mazzoni G., Coutinho L.L., Banerjee P., Geistlinger L., Cesar A.S. (2019). Detection of co-expressed pathway modules associated with mineral concentration and meat quality in Nelore cattle. Front. Genet..

[26] Pilarczyk R. (2014). Elemental Composition of Muscle Tissue of Various Beef Breeds Reared Under Intensive Production Systems. Int. J. Environ. Res..

[27] Santana A.F., Silva E., Viana Z.C.V., Korn M.G.A., Santos V.L.C.S. (2015). Avaliação de Elementos Químicos Essenciais e Chumbo em Tecidos Bovinos na Bahia, Brasil. Eciclopedia Biosf..

[28] Institute of Medicine (1997). Dietary Reference Intakes for Calcium, Phosphorus, Magnesium, Vitamin D, and Fluoride.

[29] Schonewille J.T., Klooster A.V., Wouterse H., Beynen A. (1999). Effects of intrinsic potassium in artificially dried grass and supplemental potassium bicarbonate on apparent magnesium absorption in dry cows. J. Dairy Sci..

[30] Kämpf N., Kern D.C. (2005). O solo como registro da ocupação humana pré-histórica na Amazônia. Tópicos em Ciência do solo.

[31] Glaser B. (2007). Prehistorically modified soils of central Amazônia: A model for sustainable agriculture in the twenty-first century. Philos. Trans. R. Soc..

[32] Flores B.M., Holmgren M., Xu C., Van Nes E.H., Jakovac C.C., Mesquita R.C., Scheffer M. (2017). Floodplains as an Achilles’ heel of Amazonian forest resilience. Proc. Natl. Acad. Sci. USA.

[33] Sales J., Kotrba R. (2013). Meat from wild boar (*Sus scrofa* L.): A review. Meat Sci..

[34] Tomović V., Jokanović M., Tomović M., Lazović M., Šojić B., Škaljac S., Ivić M., Kocić-Tanackov S., Tomašević I., Martinović A. (2017). Cadmium in liver and kidneys of domestic Balkan and Alpine dairy goat breeds from Montenegro and Serbia. Food Addit. Contam.-Part B.

[35] National Research Council (1976). Subcommittee on Beef Cattle Nutrition. Nutrient Requirements of Beef Cattle.

[36] Ministério da Saúde (2014). Secretaria de Atenção à Saúde. Departamento de Atenção Básica. Guia Alimentar para a População Brasileira/Ministério da Saúde, Secretaria de Atenção à Saúde, Departamento de Atenção Básica.

[37] Mendonça Júnior A.F., Braga A.P., Rodrigues A.P.M.S., Sales L.E.M., Mesquita H.C. (2011). Minerais: Impotância de uso na dieta de ruminantes. ACSA-Agropecuária Científica Semi-Árido.

[38] Patel N., Bergamaschi M., Magro L., Petrini A., Bittante G. (2019). Relationships of a detailed mineral profile of meat with animal performance and beef quality. Animals.

[39] McDowell L.R., Conrad J.H. (1977). Trace Mineral Nutrition in Latin American.

[40] McAfee A.J., McSorley E.M., Cuskelly G.J., Moss B.W., Wallace J.M., Bonham M.P. (2010). Red meat consumption: An overview of the risks and benefits. Meat Sci..

[41] Rooke J.A., Flockhart J.F., Sparks N.H. (2010). The potential for increasing the concentrations of micro-nutrients relevant to human nutrition in meat, milk and eggs. J. Agric. Sci..

[42] Mahan L.K., Escott-Stump S. (2005). Alimentos, Nutrição e Dietoterapia.

[43] Almeida C., Franco E.C.R. (2014). Curso Didático de Nutrição—Parte 1 Nutrição Humana.

[44] Cozzolino S.M.F. (2012). Biodisponibilidade de Nutrientes.

